# Supporting GPs in the Management of Children and Young People with ADHD Through Project ECHO^®^: Results from a Self-Efficacy Survey

**DOI:** 10.5334/ijic.6531

**Published:** 2022-07-06

**Authors:** Dana Newcomb, Phil Nixon, Perrin Moss, Vishal Kapoor

**Affiliations:** 1 Children’s Health Queensland, AU; 2 Primary Care Clinical Unit, Faculty of Medicine, The University of Queensland, AU; 3 School of Health and Rehabilitation Sciences, The University of Queensland, AU

**Keywords:** Project ECHO, communities of practice, virtual learning, Attention Deficit Hyperactivity Disorder, Australia, paediatrics, integration, Queensland

## Abstract

**Introduction:**

Attention Deficit Hyperactivity Disorder (ADHD) accounts for a high proportion of paediatric outpatient visits in Australia. Shared care by general practitioners (GPs) would deliver more timely care, closer to home, however GPs indicated the need for interprofessional training support. This study describes the use of Project ECHO^®^, a guided practice model, to support GPs with ADHD management, by connecting them virtually with an interprofessional team of paediatric specialists using a structured methodology.

**Methods:**

A retrospective pre/post-knowledge and self-efficacy survey across twenty-seven aspects of ADHD management was administered, using a seven-point Likert scale.

**Results:**

Significant improvement (p < 0.001) in provider self-efficacy was demonstrated across all tested domains.

**Discussion:**

Use of the ECHO model™ by an interprofessional team of paediatric specialists achieved an increase in GP knowledge and self-efficacy in the local management of children and young people with complex healthcare needs. Learnings indicate viability to expand the application of the ECHO model™ to address fragmentation for other priority populations across the Australian healthcare and human service sector landscape.

**Conclusion:**

Use of the ECHO model™ to support and train GPs was successful. Integration of care was achieved through strengthened partnerships between content and context experts, and the ECHO model™’s case-based learning methodology.

## Introduction

Attention Deficit Hyperactivity Disorder (ADHD) is a neurodevelopmental condition affecting 5–7% of Australian children and accounts for 20% of paediatric outpatient encounters nationally [1,2]. This represents a significant workload for public paediatric health services and contributes to long waiting periods for children to access new appointments. This study proposed that a virtual shared-care model with monitoring and routine prescribing in primary care following diagnosis and treatment initiation by the paediatrician could be an effective solution.

A previous study of GP attitudes around ADHD management found that GPs do not feel equipped to manage ADHD [3]. GP prescribing of scheduled or controlled drugs commonly referred to as S8s, including stimulants is also not common in Australia due to barriers including lack of training, time constraints, concerns about misuse, and variations in State and Territory regulations [3,4]. However, GPs report a willingness to increase their involvement in ADHD if provided with training and support [3,5].

To address this need for training and multidisciplinary input, Children’s Health Queensland Hospital and Health Service (CHQ) introduced a Project ECHO^®^ (Extension for Community Healthcare Outcomes) pilot to support GPs to manage children and adolescents with ADHD [6,7]. ECHO^®^ is a virtual hub-and-spoke model of collaborative, case-based learning, delivered via regular videoconferences. The ECHO model™ has been used extensively around the world to create virtual knowledge networks, or communities of practice, by incorporating learning strategies informed by theoretical frameworks including Social Cognitive Theory, Situated Learning Theory, and Community of Practice Theory [8,9,10]. ECHO^®^ connects a multidisciplinary ‘hub’ team of specialists, to ‘spoke’ primary care providers, to collaboratively discuss de-identified patient cases during regular videoconferences. The model has proven to be an effective training method for a range of complex conditions internationally [11].

The ECHO model™ adopts a core learner-centric approach to the virtual hub-and-spoke education, based on a key principle of “all teach and all learn” [12]. Specialist teams at the ‘hub’ site mentor primary care providers and other frontline professionals, including general practitioners (GPs), nursing, allied health, and other health/human services professionals at ‘spokes’, and all participants learn from one another’s expertise and insights [12,13]. Spoke participants are encouraged to share their deep knowledge of local social and cultural considerations, and an understanding of realistic approaches to care within their specific communities [14]. The specialists offer complementary content expertise, and over time virtual ‘communities of practice’ or ‘knowledge networks’ develop whereby each participant plays a role in co-producing the knowledge and developing the skills to manage complex conditions [8]. Globally there are hundreds of organisations using the ECHO model™ to provide a virtual solution to enhance and support shared care by empowering local frontline providers to deliver care closer to home with timely support from multidisciplinary teams of specialists [10,15,16,17,18].

In January 2016, the then Queensland Minister for Health and Minister for Ambulance Services commissioned a $35 million-dollar Integrated Care Innovation Fund (ICIF) [6]. The fund sought to stimulate new and collaborative integrated care proposals from Queensland’s 16 state-funded Hospital and Health Services (HHSs) in partnership with federally-funded Primary Health Networks (PHNs). HHSs are statutory bodies established to operate as the principal providers of public sector hospital and health services across Queensland [19]. PHN organisations function to increase efficiency and effectiveness of primary care services for patients, particularly those at risk of poor health outcomes, by improving coordination of care that ensures provision of the right care in the right place at the right time [20]. PHNs were designed to achieve these objectives by working directly with general practitioners, other primary, secondary and tertiary healthcare providers [20].

The CHQ research team applied and was successful in obtaining an ICIF grant to pilot the ECHO model™ for the first time in Queensland to support GPs with ADHD management [7]. The basis for this grant was to establish and resource an ECHO^®^ hub at CHQ, based in South Brisbane, and to launch and evaluate the pilot focussed on ADHD. The grant term also committed that CHQ would work in partnership with two local PHNs throughout the grant term (2016–2018) to raise awareness and promote the ADHD ECHO^®^ pilot to primary care audiences.

The aim of this study was to investigate whether the use of the ECHO model™ to support and train GPs was successful through a self-efficacy survey. The change in the integration of care was assessed based on GPs perceptions of the established partnerships between content and context experts, and the ECHO^®^ model™’s case-based learning methodology.

## Ethical Approval

This study was approved by the Children’s Health Queensland Hospital and Health Service Human Research Ethics Committees (Ref: HREC/17/QRCH/67).

## Methods

### ECHO^®^ Hub Establishment

CHQ established a Project ECHO^®^ hub as a collaborative model of education and case-based discussions to allow paediatric experts to share knowledge with GPs and other healthcare professionals in the primary care sector. Education was delivered via a series of weekly videoconference sessions from April 2017 with a panel of experts located at a central ‘hub’ site, while providers remained in their local setting. CHQ leveraged the virtual nature of the ECHO model™ to support primary healthcare providers in urban, regional, rural, and remote settings to better manage patients with an ADHD diagnosis and complex healthcare needs. In each of these geographic settings, access to specialist services was limited. In this project, the ECHO^®^ series were targeted at empowering GPs to better manage children with ADHD.

### Project Team Training

The CHQ project team consisted of a General Practice Liaison Officer, Project Manager, and General Paediatrician – with each key staff member responsible for aspects of the planning and implementation of the pilot. A crucial step in the initial planning phase involved sending the project team to complete the ECHO Institute™’s Immersion training at the University of New Mexico, USA. Immersion training is compulsory for prospective hub organisations to become licensed to use the ECHO model™ and provided the team with extensive training in the ECHO model™.

### Governance and Stakeholder Engagement

The project team worked to gain and maintain organisational support and buy-in from key executive leaders in Queensland. Stakeholders, including representatives from CHQ, PHNs, the Department of Health’s Clinical Excellence Queensland (CEQ), GPs, and a consumer representative, were engaged to form a project steering committee that was responsible for key decision-making throughout the pilot. This governance forum oversaw and endorsed all aspects of the pilot throughout the implementation phase. The project team designed a locally-responsive curriculum for the first paediatric pilot ECHO^®^ series in Queensland based on a literature review, example curricula from another ECHO^®^ hub site delivering an ADHD ECHO^®^ Network, and through consultation with ADHD subject matter experts. Subsequently, a multidisciplinary panel of experts was identified and engaged to facilitate the ADHD ECHO^®^ Network over several cohort cycles known as series. Stakeholder engagement was maintained throughout the implementation through project governance meetings and targeted promotional activities on social media. Project staff networked effectively to engage new stakeholders and panellist expertise where necessary throughout the implementation period.

### Delivering the pilot ADHD ECHO^®^ Network

The project team utilised a variety of methods to market the training and recruit participants. Back-to-back ADHD ECHO^®^ Network series were delivered in line with the Queensland school term calendar. GPs interested in learning more about ADHD were recruited to join the ADHD ECHO^®^ Network, through marketing support provided by the Queensland PHNs. There was no requirement for the GPs to have previous experience managing patients with ADHD. In addition to GPs, participation was open to GP registrars, psychologists, nurses, and paediatric trainees. Feedback was collected from various stakeholders and participants, allowing for adjustments and improvements throughout implementation.

Cohorts of up to 20 participants joined weekly, 90-minute videoconferences for 10-week cohorts throughout 2017–2018. Each ECHO^®^ session commenced with a brief didactic presentation by a hub panellist on an aspect of ADHD management identified in the curriculum. Following this, participants presented de-identified cases to peers and the hub panel as a virtual team. Throughout the didactic and case presentation components of the ECHO^®^ session, all participants had the opportunity for raising questions for clarification and offering evidence-based recommendations. The ADHD ECHO^®^ panel was made up by a developmental paediatrician, psychologist, senior guidance officer, general paediatrician, and parent representative. The parent representative, recruited through ADHD Australia, brought rich insight and lived experience raising four children with ADHD and other behavioural health needs [21,22]. Participants retained full responsibility for their patient case presentations and could choose whether to incorporate the advice received during ECHO^®^ Network sessions into their local patient management. Participants were encouraged to re-present their patients at subsequent ECHO^®^ sessions if required for further advice and support.

Continuing Professional Development (CPD) points for attending the ADHD ECHO^®^ Network were accredited by the Royal Australian College of General Practitioners (RACGP) and/or Australian College of Rural and Remote Medicine (ACRRM). The CPD points were offered to GPs who completed the pre-disposing activity, attended a minimum of six ECHO^®^ sessions, and completed the evaluation including retrospective pre/post-ECHO^®^ self-efficacy survey. Other professional disciplines that participated in the ECHO^®^ sessions were able to self-report the CPD activity to their relevant professional bodies and were provided with attendance transcripts for evidence.

Following each of the five 10-week series, all participants were invited to complete the retrospective pre/post-ECHO^®^ self-efficacy survey (Appendix 1) across twenty-seven aspects of ADHD management including symptom monitoring, prescription of stimulants, assessment and management of co-morbidities, and referral to allied health and school support services [1,5,23,24,25,26,27,28]. A Likert scale from 1–7 was used for each item, with terminal anchors of “no confidence” and “very confident”. Only GP participants who met the requirements for awarding of CPD points were included in this analysis.

## Results

Once CHQ’s ECHO^®^ hub was successfully established, five ADHD ECHO^®^ Network series were delivered to a total of 65 primary care providers, including 43 GPs. During the study period, 31 out of 43 GP participants from five of the ADHD ECHO^®^ Network cohorts (total N = 43; response rate: 72.1%) responded to the retrospective pre/post- ECHO^®^ self-efficacy survey. Data were analysed using Stata 15.1 software [29]. The Likert scale responses were compared using the non-parametric Wilcoxon paired sign-rank test. The eligibility criteria were set as participants attending a minimum of six ECHO^®^ sessions to meet the RACGP and ACCRM CPD awarding requirements. Other respondents who did not meet the minimum of attending six ECHO^®^ sessions were excluded from this data set.

**Figure 1:**
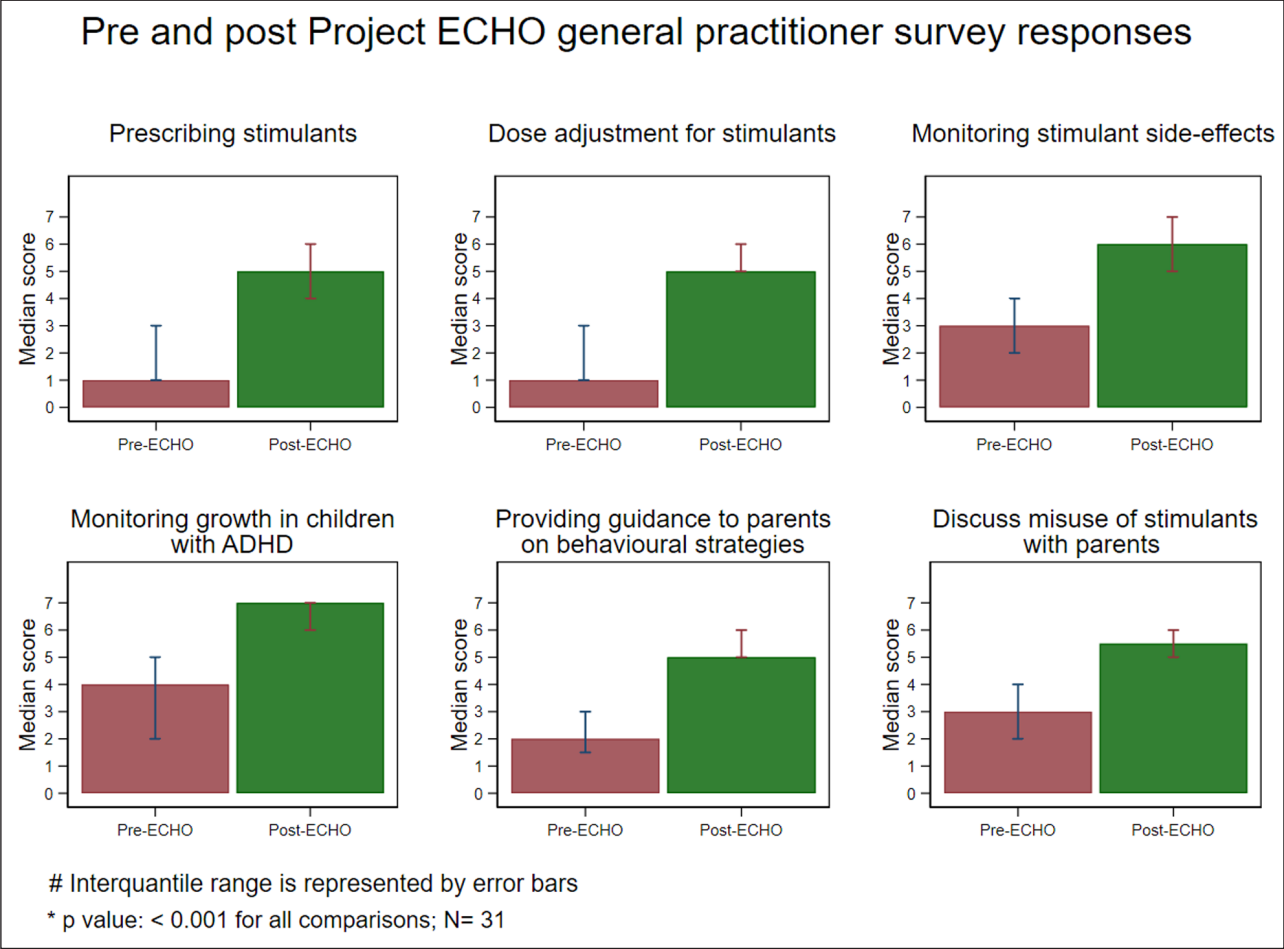
Pre and post Project ECHO^®^ general practitioner survey responses.

There was statistically significant difference (p value < 0.001) in the pre/post-ECHO^®^ survey responses in all surveyed domains (Figure 1). Importantly the maximum absolute magnitude of effect was demonstrated in the prescription of stimulants, dose adjustment for stimulants, weaning stimulants, changing from short to long-acting stimulants and assessment of anxiety (median score difference of 4 in the Likert scale between pre- and post- responses). Figure 1 below illustrates the pre- and post-ECHO^®^ self-efficacy results for the 31 GPs who met the inclusion criteria.

This data provided evidence that the innovation improved GPs’ capacity and confidence to manage children with ADHD in primary care. In addition, the research team acknowledged that there were challenges experienced by the project team in sustaining rates of GP participation throughout the pilot. Anecdotally, these challenges were in part attributable to the funding instrument whereby the federal government funds a fee-for-service model in the Australian primary care setting which discourages GPs from participating in learning opportunities during income generating business hours [30,31,32]. Future studies are warranted to analyse longer term data on the ability of ECHO^®^-trained GPs to manage patients in the primary care setting to determine if impacts on patient outcomes and cost-effectiveness of using the ECHO model™ in the Australian context are similar to international experiences [11,33,34].

*Did the innovation result in improved GP knowledge and confidence in the diagnosis and management of children with ADHD?*

GP participants rated their own self-efficacy across 27 dimensions related to the management of children with ADHD [1,5,23,24,25,26,27,28]. The questionnaire was completed by a total of 31 GP participants, out of the total 43 GPs who were involved throughout the five cohorts. Figure 1 above presented the GP respondent results.

The survey was completed retrospectively for collecting both pre- and post-ECHO^®^ Network responses in line with guidance from the ECHO Institute™, University of New Mexico Health Sciences Center. This was to reduce the risk of biasing in pre-ECHO^®^ self-efficacy responses if completed prior to attending the sessions, whereby participants may score their knowledge and confidence higher pre-ECHO^®^, despite generally “not knowing what they don’t know”. Strong improvements were seen in participant self-efficacy across all 27 dimensions. On average, self-efficacy scores increased from 2.6 to 5.4 on a seven-point scale.

*The strongest improvements in GP self-efficacy were observed in responses to*:

- Switch from short acting to long-acting stimulants (pre-ECHO^®^: 1.6, post-ECHO^®^: 5.1);
- Use the Vanderbilt questionnaire to monitor symptoms of a child with ADHD (pre-ECHO^®^: 1.0, post-ECHO^®^: 2.9);
- Prescribe stimulants to children with ADHD (pre-ECHO^®^: 1.7, post-ECHO^®^: 5.0);
- Serve as a ‘local consultant’ within your clinic or community for ADHD questions (pre-ECHO^®^: 1.7, post-ECHO^®^: 4.9);
- Discuss with families what educational support is available for children with ADHD (pre-ECHO^®^: 1.9, post-ECHO^®^: 5.6).

*The highest overall level of GP self-efficacy upon completion of an ADHD ECHO^®^ series was observed in responses to*:

- Monitor growth in children with ADHD taking stimulant medication (6.5);
- Monitor blood pressure in children with ADHD taking stimulant medication (6.2);
- Know when a referral back to a paediatrician may be required (6.1);
- Refer a child to a school guidance officer (5.9);
- Screen for side effects of stimulant medication in children with ADHD (5.8).

## Discussion

This study is the first to examine changes in Australian GP self-efficacy in the management of paediatric ADHD, after participating in an ECHO^®^ Network. The study provides proof of concept that the ECHO™ model can be used to empower and support GPs in ADHD management, and that a virtual shared-care model could feasibly be implemented. Increased virtual shared-care of young people with ADHD could potentially ameliorate the rising demand for paediatric outpatient ADHD care [4,35], and facilitate delivery of more timely care, closer to home by primary care providers such as GPs. This is particularly evident when noting that the highest increase in the median scores occurred in domains related to prescription and dose adjustment of stimulants, an important aspect of any future shared-care model between primary and secondary/tertiary healthcare providers in Australia.

Limitations of the study included both the potential for recall and response bias due to the retrospective and self-reporting nature of survey responses, and the small number of survey responses. The researchers also acknowledge that the GPs who participated in this study were volunteers, and there was no comparison group, so desirability bias cannot be excluded.

Future research should be conducted to determine if the increased self-efficacy observed in this study is sustained long-term. These results also warrant further research comparing health outcomes and consumer satisfaction with care of patients who are cared for by ECHO^®^-trained GPs, with outcomes of patients receiving ‘usual care’ in paediatric outpatient department settings. Research into the longer-term cost-effectiveness of GP training via ECHO^®^ in Australia is also required.

This research demonstrates scalable learnings that can support the GP workforce through wider use of the ECHO model™ being replicated in a variety of different topic areas and across other professional, sectoral, and geographic jurisdictions. The ADHD ECHO^®^ pilot presents a compelling example to system managers at state and federal levels, of an innovative way to virtually share care for patients with complex healthcare needs across the primary-secondary-tertiary continuum and beyond geographic boundaries.

### Barriers to GP Recruitment

The researchers acknowledge the inherent limitations within the intervention design to sustain ongoing participation of GP participants in the ADHD ECHO^®^ Network. Some barriers were structural, relating to external factors that would be difficult to resolve. Others were internal, relating to some of the prescriptive aspects of the ECHO model™ and session structure, and the potential mismatch with the Australian primary care funding model.

While spoke participation in all ECHO^®^ Network sessions are mandated to always be free, GPs can also be eligible to receive CPD points for participation in any accredited ECHO^®^ Network. If a GP presents a patient case in any ECHO^®^ Network session, they can self-determine their eligibility to claim a case-conferencing item number under the Medicare Benefits Scheme. This would only serve to partially remunerate the GPs lost income-generating time at ECHO^®^ Network sessions in which they presented a patient case from their own practice for advice and support. Despite these characteristics of the ECHO^®^ Network intervention, it remains unclear whether the provision of CPD points alone would remain a sufficient incentive to attract and retain a solid, steady flow of GPs to enrol in the ECHO^®^ Network longer-term.

The prescribed nature of the ECHO model™’s virtual delivery method and regular timeframe constrained the number of GPs able and willing to participate in the ADHD ECHO^®^ Network. Participation required a one-hour per week commitment over 10 weeks at a specified time to be eligible for the maximum CPD points. ECHO^®^ Network participation during business hours would typically result in a GP losing revenue, due to the fee-for-service funding model and many GPs not having a salary retainer. This was seen as unattractive for many potential GP participants, and unacceptable for some.

The Australian landscape of CPD provision is competitive, with a variety of options constantly marketed to GPs by professional colleges for upskilling, was also identified as a barrier to uptake. Several strategies were explored, however a highly effective method of marketing the ADHD ECHO^®^ Network to the target population of participants was yet to be identified. The associated workload for the project team in registering and onboarding GPs was identified as resource intensive and future planning to streamline this should be investigated.

It is hypothesised that as the ECHO model™’s use in Australia expands, its reputation as a well-known, high-quality option for primary care professional development will increase. This would complement and offset some of the project team’s efforts during the pilot to gain GP participant enrolments. Based on enrolments across the variety of CHQ’s ECHO^®^ Networks after the ADHD ECHO^®^ pilot, there is also growing evidence that this method of learning appeals to a wide array of other professional groups.

### Costs and Incentives for ECHO^®^ Panellists

For the initial ADHD ECHO^®^ Network pilot, CHQ employees who had ECHO^®^ panellist roles were funded through the ICIF grant to enable the backfilling of their usual roles. This was to ensure continuity of frontline services while this pilot was being tested.

However, in subsequent series beyond the grant term of the ADHD ECHO^®^ Network pilot, the project team relied on in-kind support from a variety of internal and external colleagues to fulfill panellist roles. Organisational incentives to serve as panellists were utilised such as professional networking and knowledge-sharing with new colleagues at spoke sites to attract content experts from across CHQ and other specialist partner agencies. This was generally straightforward and sustainable as organisational role descriptions commonly required more senior staff to commit to providing education and training as a core responsibility. The sustainability of this aspect of the ECHO model™ in particular for external staff with subject matter expertise needs to be considered.

### Cultural Barriers

Over the course of the ADHD ECHO^®^ Network pilot, it was discovered that even when the innovation was successful in improving the confidence and capacity of GPs to manage children with behavioural conditions such as ADHD, there were cultural barriers to changing engrained practice with the potential to prevent and/or reduce the bottleneck service burden of the HHS. Historically, GPs in Queensland have tended not to use the prescribing delegations they have to treat children with ADHD.

While a residual culture of hospital staff not trusting GPs in the community enough to discharge patients to their care remains, positive changes were reported amongst the paediatric specialists involved in the delivery the ADHD ECHO^®^ Network series regarding their GP colleagues.

This research provides evidence to suggest the Project ECHO^®^ innovation may achieve an increase in paediatricians’ discharge of ADHD patients to general practice following diagnosis and initiation of treatment. GPs who were interviewed reported that participating in the ADHD ECHO^®^ Network series resulted in improvements in their self-efficacy and capacity to manage ADHD patients without needing to refer to a paediatrician.

It is likely that a significant reduction in new referrals from GPs who had completed the ADHD ECHO^®^ Network series would be seen over time and warrants further research over a longer-term period. This was confirmed by the GPs surveyed who reported that participation in the ADHD ECHO^®^ Network series might decrease the future chance of needing to refer patients to a paediatrician. As a result of their participation in the ADHD ECHO^®^ Network, some GPs reported that they developed collegial relationships with the paediatric panellists and other GP spoke participants, such that if faced with a difficult patient, the GP was inclined to first contact one of those now-known colleagues for advice before deciding to refer to a hospital outpatient service. A reduction in health service utilisation and costs due to improved local capacity for ADHD management in primary care would therefore be feasible but would require longer term data to validate. Consideration of similar benefits across state and federally-funded health systems should be explored where the ECHO model™ could be applied to enable more virtual shared care solutions to system pressures.

## Lessons Learned

- It takes time to achieve genuine culture change and reduce historical/hierarchical/professional barriers;
- Primary care fee-for-service funding models inhibit the GP workforce from engaging in ECHO^®^ Networks, despite the international evidence linking the ECHO model™ of virtual shared care to improved outcomes;
- Participation in ECHO^®^ Networks can improve GP knowledge and self-efficacy;
- The use of the ECHO model™ in Australia can successfully integrate care.

## Conclusion

The aim of this study was to investigate whether the use of the ECHO model™ to support and train GPs was successful through a self-efficacy survey. The change in the integration of care was assessed based on GPs perceptions of the established partnerships between content and context experts, and the ECHO model™’s case-based learning methodology.

This study found that the use of the ECHO model™ to support and train GPs in managing children with ADHD was successful. The improved integration of care was achieved through strengthened partnerships between content and context experts, and the ECHO model™’s case-based learning methodology.

## Appendix

**Appendix 1:** ADHD ECHO^®^ Self-Efficacy Survey.

ADHD ECHO^®^ NETWORK PRE/POST SELF-EFFICACY								

How CONFIDENT are you in your ABILITY to EFFECTIVELY: 1=no confidence 7 = very confident								

A. Royal Australian College of General Practitioners No:								

B. Australian College of Rural and Remote Medicine No:								

1. Use the Vanderbilt questionnaire to monitor symptoms of a child with ADHD	After Series	1	2	3	4	5	6	7

	Before Series	1	2	3	4	5	6	7

2. Guide parents in the use of common behavioural strategies for managing challenging behaviours in children with ADHD	After Series	1	2	3	4	5	6	7

	Before Series	1	2	3	4	5	6	7

3. Monitor blood pressure in children with ADHD taking stimulant medication	After Series	1	2	3	4	5	6	7

	Before Series	1	2	3	4	5	6	7

4. Monitor growth in children with ADHD taking stimulant medication	After Series	1	2	3	4	5	6	7

	Before Series	1	2	3	4	5	6	7

5. Screen for side effects of stimulant medication in children with ADHD	After Series	1	2	3	4	5	6	7

	Before Series	1	2	3	4	5	6	7

6. Prescribe stimulants to children with ADHD	After Series	1	2	3	4	5	6	7

	Before Series	1	2	3	4	5	6	7

7. Change the dose of stimulants for behavioural escalation or change in weight	After Series	1	2	3	4	5	6	7

	Before Series	1	2	3	4	5	6	7

8. Switch from short acting to long-acting stimulants	After Series	1	2	3	4	5	6	7

	Before Series	1	2	3	4	5	6	7

9. Decrease the stimulant dose or stop stimulants according to clinical context	After Series	1	2	3	4	5	6	7

	Before Series	1	2	3	4	5	6	7

10. Discuss with families what educational support is available for children with ADHD	After Series	1	2	3	4	5	6	7

	Before Series	1	2	3	4	5	6	7

11. Help families access allied health therapy for children with ADHD	After Series	1	2	3	4	5	6	7

	Before Series	1	2	3	4	5	6	7

12. Identify parent support resources for parents of children with ADHD	After Series	1	2	3	4	5	6	7

	Before Series	1	2	3	4	5	6	7

13. Refer a child to a school guidance officer	After Series	1	2	3	4	5	6	7

	Before Series	1	2	3	4	5	6	7

14. Know when a referral back to a paediatrician may be required	After Series	1	2	3	4	5	6	7

	Before Series	1	2	3	4	5	6	7

15. Assess and manage sleep problems in children with ADHD (whether or not on stimulant medication)	After Series	1	2	3	4	5	6	7

	Before Series	1	2	3	4	5	6	7

16. Assess and manage constipation in children with ADHD	After Series	1	2	3	4	5	6	7

	Before Series	1	2	3	4	5	6	7

17. Assess and manage appetite and dietary intake in children with ADHD	After Series	1	2	3	4	5	6	7

	Before Series	1	2	3	4	5	6	7

18. Assess for co-morbid anxiety in children with ADHD	After Series	1	2	3	4	5	6	7

	Before Series	1	2	3	4	5	6	7

19. Discuss treatment options for co-morbid anxiety in children with ADHD	After Series	1	2	3	4	5	6	7

	Before Series	1	2	3	4	5	6	7

20. Assess for mood disorder in children with ADHD	After Series	1	2	3	4	5	6	7

	Before Series	1	2	3	4	5	6	7

21. Discuss treatment options for mood disorder in children with ADHD	After Series	1	2	3	4	5	6	7

	Before Series	1	2	3	4	5	6	7

22. Answer parental questions about ADHD and CAM (Complementary and Alternative Medicine)	After Series	1	2	3	4	5	6	7

	Before Series	1	2	3	4	5	6	7

23. Answer parental questions about ADHD and puberty/growth	After Series	1	2	3	4	5	6	7

	Before Series	1	2	3	4	5	6	7

24. Provide appropriate anticipatory guidance to families raising children with ADHD	After Series	1	2	3	4	5	6	7

	Before Series	1	2	3	4	5	6	7

25. Partner with parents to facilitate decision making about their child	After Series	1	2	3	4	5	6	7

	Before Series	1	2	3	4	5	6	7

26. Discuss your concerns about misuse of stimulant medication with parents	After Series	1	2	3	4	5	6	7

	Before Series	1	2	3	4	5	6	7

27. Serve as a ‘local consultant’ within your clinic or community for ADHD questions	After Series	1	2	3	4	5	6	7

	Before Series	1	2	3	4	5	6	7

## References

[1] Skounti M, Philalithis A, Galanakis E. Variations in prevalence of attention deficit hyperactivity disorder worldwide. Eur J Pediatr. 2007; 166(2): 117–23. DOI: 10.1007/s00431-006-0299-5 17033803

[2] Hiscock H, Roberts G, Efron D, Sewell JR, Bryson HE, Price AMH, et al. Children Attending Paediatricians Study: a national prospective audit of outpatient practice from the Australian Paediatric Research Network. Med J Aust. 2011; 194(8): 392–7. DOI: 10.5694/j.1326-5377.2011.tb03028.x 21495938

[3] Shaw KA, Mitchell GK, Wagner IJ, Eastwood HL. Attitudes and practices of general practitioners in the diagnosis and management of attention-deficit/hyperactivity disorder. J Paediatr Child Health. 2002; 38(5): 481–6. DOI: 10.1046/j.1440-1754.2002.00033.x 12354265

[4] Pharmaceutical Benefits Scheme. ADHD: utilisation analysis: Department of Health; 2015. Available from: http://www.pbs.gov.au/info/industry/listing/participants/public-release-docs/2015-06/attention-deficit-hyperactivity-disorder-2015-06-prd.

[5] Shaw K, Wagner I, Eastwood H, Mitchell G. A qualitative study of Australian GPs’ attitudes and practices in the diagnosis and management of attention-deficit/hyperactivity disorder (ADHD). Family Practice. 2003; 20(2): 129–34. DOI: 10.1093/fampra/20.2.129 12651785

[6] Mundy L, Hewson K. Thinking outside the system: the integrated care experience in Queensland, Australia. Australian Journal of Primary Health. 2019; 25(4): 303–9. DOI: 10.1071/PY18161 31439125

[7] Moss P, Hartley N, Ziviani J, Newcomb D, Russell T. Executive Decision-Making: Piloting Project ECHO to Integrate Care in Queensland. International Journal of Integrated Care. 2020; 20(4): 1–15. DOI: 10.5334/ijic.5512 PMC7716780 33335464

[8] Socolovsky C, Masi C, Hamlish T, Aduana G, Arora S, Bakris G, et al. Evaluating the Role of Key Learning Theories in ECHO: A Telehealth Educational Program for Primary Care Providers. Progress in Community Health Partnerships. 2013; 7(4): 357–8. DOI: 10.1353/cpr.2013.0052 24375176

[9] Wenger E, McDermott R, Snyder W. Cultivating communities of practice: a guide to managing knowledge. Boston: Harvard Business School Press; 2002.

[10] ECHO Institute. ECHO hubs Albuquerque: University of New Mexico Health Sciences Center; 2020. Available from: https://echo.unm.edu/locations.

[11] Zhou C, Crawford A, Serhal E, Kurdyak P, Sockalingam S. The Impact of Project ECHO on Participant and Patient Outcomes: A Systematic Review. Acad Med. 2016; 91(10): 1439–61. DOI: 10.1097/ACM.0000000000001328 27489018

[12] ECHO Institute. Project ECHO Albuquerque: University of New Mexico Health Sciences Centre; 2020 [cited 11 February 2020]. Available from: https://echo.unm.edu/.

[13] Arora S, Kalishman S, Thornton K, Dion D, Murata G, Deming P, et al. Expanding access to hepatitis C virus treatment—Extension for Community Healthcare Outcomes (ECHO) project: Disruptive innovation in specialty care. Hepatology. 2010; 52(3): 1124–33. DOI: 10.1002/hep.23802 20607688 PMC3795614

[14] Arora S, Kalishman S, Dion D, Som D, Thornton K, Bankhurst A, et al. Partnering urban academic medical centers and rural primary care clinicians to provide complex chronic disease care. Health Affairs (Project Hope). 2011; 30(6): 1176–84. DOI: 10.1377/hlthaff.2011.0278 21596757 PMC3856208

[15] Brandt L, Despins L, Wakefield B, Fleming D, Deroche C, Popejoy L. Health care ethics ECHO: Improving ethical response self-efficacy through sensemaking. International Journal of Ethics Education. 2021; 6(1): 125–39. DOI: 10.1007/s40889-021-00119-1

[16] De Witt Jansen B, Brazil K, Passmore P, Buchanan H, Maxwell D, McIlfatrick SJ, et al. Evaluation of the impact of telementoring using ECHO© technology on healthcare professionals’ knowledge and self-efficacy in assessing and managing pain for people with advanced dementia nearing the end of life. BMC Health Serv Res. 2018; 18(1): 228-. DOI: 10.1186/s12913-018-3032-y 29606132 PMC5879835

[17] Furlan AD, Zhao J, Voth J, Hassan S, Dubin R, Stinson JN, et al. Evaluation of an innovative tele-education intervention in chronic pain management for primary care clinicians practicing in underserved areas. Journal of Telemedicine and Telecare. 2019; 25(8): 484–92. DOI: 10.1177/1357633X18782090 29991316

[18] Goldman MP, Auerbach MA, Garcia AM, Gross IT, Tiyyagura GK. Pediatric Emergency Medicine ECHO (Extension for Community Health Care Outcomes): Cultivating Connections to Improve Pediatric Emergency Care. AEM Education and Training. 2020; 0(1–6). DOI: 10.1002/aet2.10548 PMC8164662 34141996

[19] Hospital and Health Boards Act, Queensland (2011).

[20] Department of Health. PHN Background. Canberra. 2018. Available from: https://www1.health.gov.au/internet/main/publishing.nsf/Content/PHN-Background.

[21] Greever-Rice T, Brandt L, Warne-Griggs M, Hoffman K. Integrating the Lived Experience Conditions and Care in the ECHO Model. Mo Med. 2020; 117(3): 241–4. 32636557 PMC7302038

[22] Moss PW, Dunlop E. Living the values – respect, integrity, care and imagination: Investing in co-design to pave the way for consumers to be project partners in paediatric health service innovation. International Journal of Integrated Care. 2018; 18(S1): 84. DOI: 10.5334/ijic.s1084

[23] Thomas R, Mitchell G, Batstra L. Attention-deficit/hyperactivity disorder: are we helping or harming? British Medical Journal. 2013; 347(7932): 18. DOI: 10.1136/bmj.f6172 24192646

[24] Wilens TE, Spencer TJ. Understanding attention-deficit/hyperactivity disorder from childhood to adulthood. Postgrad Med. 2010; 122(5): 97–109. DOI: 10.3810/pgm.2010.09.2206 20861593 PMC3724232

[25] Wolraich ML, Hagan JF, Jr, Allan C, Chan E, Davison D, Earls M, et al. Clinical Practice Guideline for the Diagnosis, Evaluation, and Treatment of Attention-Deficit/Hyperactivity Disorder in Children and Adolescents. Pediatrics. 2019; 144(4). DOI: 10.1542/peds.2019-2528 PMC7067282 31570648

[26] Wolraich ML, Lambert W, Doffing MA, Bickman L, Simmons T, Worley K. Psychometric properties of the Vanderbilt ADHD Diagnostic Parent Rating Scale in a referred population. J Pediatr Psychol. 2003; 28(8): 559–67. DOI: 10.1093/jpepsy/jsg046 14602846

[27] American Psychiatric Association DSMTF. Diagnostic and statistical manual of mental disorders: DSM-5. 5th ed. Washington, DC & Arlington, VA: American Psychiatric Association; 2013. DOI: 10.1176/appi.books.9780890425596

[28] National Institute for Children’s Health Quality. NICHQ Vanderbilt Assessment Scales. 2002.

[29] StataCorp LLC. Stata Statistical Software. Release 16 ed. Texas, USA. 2019.

[30] Nicholson C, Jackson C, Marley J. Best practice integrated primary/secondary health care governance – applying evidence to Australia’s health reform agenda. BMC Health Services Research. 2014; 14(Suppl 2): O6–O. DOI: 10.1186/1472-6963-14-S2-O6 25047885

[31] Nicholson S, Cleland JA. “It’s making contacts”: notions of social capital and implications for widening access to medical education. Adv Health Sci Educ Theory Pract. 2017; 22(2): 477–90. DOI: 10.1007/s10459-016-9735-0 27844179

[32] Jackson CL, Donald M, Russell AW, McIntyre HD. Establishing a new model of integrated primary and secondary care based around general practice: A case study of lessons learned and challenges. Aust Health Rev. 2018; 42(3): 299–302. DOI: 10.1071/AH16147 28483036

[33] Arora S, Thornton K, Murata G, Deming P, Kalishman S, Dion D, et al. Outcomes of Treatment for Hepatitis C Virus Infection by Primary Care Providers. The New England Journal of Medicine. 2011; 364(23): 2199–207. DOI: 10.1056/NEJMoa1009370 21631316 PMC3820419

[34] McBain R, Sousa J, Rose A, Baxi S, Faherty L, Taplin C, et al. Impact of Project ECHO Models of Medical Tele-Education: a Systematic Review. Journal of General Internal Medicine. 2019; 34(12): 2842–57. DOI: 10.1007/s11606-019-05291-1 31485970 PMC6854140

[35] National Health and Medical Research Council. Attention Deficit Hyperactivity Disorder. Canberra: Australian Government Publishing Service; 1996.

